# The convergence analysis of P-type iterative learning control with initial state error for some fractional system

**DOI:** 10.1186/s13660-017-1302-6

**Published:** 2017-01-31

**Authors:** Xianghu Liu, Yanfang Li

**Affiliations:** 1 Department of Mathematics, Guizhou University, Huaxi Road, Guiyang, China; 2 Mathematics Department, Zunyi Normal College, Wujiang Road, Zunyi, 563006 China

**Keywords:** 93C10, 93C40, Caputo fractional derivative, iterative learning control, convergence, Mittag-Leffler function

## Abstract

In this paper, the convergence of iterative learning control with initial state error for some fractional equation is studied. According to the Laplace transform and the M-L function, the concept of mild solutions is showed. The sufficient conditions of convergence for the open and closed P-type iterative learning control are obtained. Some examples are given to illustrate our main results.

## Introduction

In this paper, we will analysis the convergence of iterative learning control initial state error of the following fractional system:

1 $$ \left \{ \textstyle\begin{array}{l} {}^{\mathrm{c}}\mathrm{D}^{\alpha}_{t} x(t)=Ax(t)+Bu(t),\quad t\in J=[0,b], \\ x(0)=x_{0}, \\ y(t)=Cx(t), \end{array}\displaystyle \right . $$

where ${}^{\mathrm{c}}\mathrm{D}^{\alpha}_{t}$ denotes the Caputo fractional derivative of order *α*, $0<\alpha<1$. $A, B,C\in R^{n\times n}$, $u(t)$ is a control vector.

Iterative learning control (ILC) was shown by Uchiyama in 1978 (in Japanese), but only few people noticed it, Arimoto *et al.* developed the ILC idea and studied the effective algorithm until 1984, they made it to be the iterative learning control theory, more and more people paid attention to it.

The fractional calculus and fractional difference equations have attracted lots of authors during in the past years, they published some outstanding work [1–12], because they described many phenomena in engineering, physics, science, and controllability. The work of fractional order systems in iterative learning control appeared in 2001, and extensive attention has been paid to this field and great progress has been made in the following 15 years [13–19], many fractional nonlinear systems were researched [20–23]. To our knowledge, it has not been studied very extensively. In the study of iterative control theory, assume that the initial state of each run is on the desired trajectory, however, the actual operation often causes some error from the iterative initial state to the desired trajectory, so we consider the system (1) and study the convergence of the learning law.

Motivated by the above mentioned works, the rest of this paper is organized as follows: In Section 2, we will show some definitions and preliminaries which will be used in the following parts. In Sections 3 and 4, we give some results for P-type ILC for some fractional system. In Section 5, some simulation examples are given to illustrate our main results.

In this paper, the norm for the *n*-dimensional vector $w=(w_{1},w_{2},\ldots ,w_{n})$ is defined as $\|w\|=\max_{1 \leq i \leq n }|w_{i}|$, and the *λ*-norm is defined as $\|x\|_{\lambda}=\sup_{ t\in[0,T]}\{ e^{-\lambda t}|x(t)|\}$, $\lambda>0$.

## Some preliminaries for some fractional system

In this section, we will give some definitions and preliminaries which will be used in the paper, for more information, one can see [1–4].

### Definition 2.1

The integral

$$I^{\alpha}_{t}f(t)=\frac{1}{\Gamma(\alpha)} \int^{t}_{0}(t-s)^{\alpha -1}f(s)\,ds,\quad \alpha>0, $$

is called the Riemann-Liouville fractional integral of order *α*, where Γ is the gamma function.

For a function $f(t)$ given in the interval $[0,\infty)$, the expression

$${}^{\mathrm{L}}D^{\alpha}_{t}f(t)=\frac{1}{\Gamma(n-\alpha)}\biggl( \frac {d}{dt}\biggr)^{n} \int^{t}_{0}(t-s)^{n-\alpha-1}f(s)\,dt, $$

where $n=[\alpha]+1$, $[\alpha]$ denotes the integer part of number *α*, is called the Riemann-Liouville fractional derivative of order $\alpha>0$.

### Definition 2.2

Caputo’s derivative for a function $f:[0,\infty)\rightarrow R $ can be written as

$${}^{\mathrm{c}}D^{\alpha}_{t}f(t)= {}^{\mathrm{L}}D^{\alpha}_{t} \Biggl[f(t)-\sum_{k=0}^{n-1} \frac{t^{k}}{k!}f^{(k)}(0)\Biggr], \quad n=[\alpha]+1, $$

where $[\alpha]$ denotes the integer part of real number *α*.

### Definition 2.3

The definition of the two-parameter function of the Mittag-Leffler type is described by

$$E_{\alpha,\beta}(z)=\sum_{k=0}^{\infty} \frac{z^{k}}{\Gamma(\alpha k+\beta)},\quad\alpha>0,\beta>0,z\in C, $$

if $\beta=1$, we get the Mittag-Leffler function of one parameter,

$$E_{\alpha}(z)=\sum_{k=0}^{\infty} \frac{z^{k}}{\Gamma(\alpha k+1)}. $$

Now, according to [24–29], we shall give the following lemma.

### Lemma 2.4

*The general solution of equation* (1) *is given by*

2 $$\begin{aligned}& x(t)= S_{\alpha,1}(A,t)x_{0}+ \int_{0}^{t}S_{\alpha,\alpha}(A,t-s)Bu(s)\,ds, \\& S_{\alpha,\beta}(A,t)=\sum_{k=0}^{\infty} \frac{A^{k}t^{\alpha k+\beta -1}}{\Gamma(\alpha k+\beta)}. \end{aligned}$$

### Lemma 2.5

*From Definition *2.4 *in* [30], *we know that the operators* $S_{\alpha,1}(t)$, $S_{\alpha,\alpha}(t)$, $S_{\alpha,\alpha-1}(t)$ *are exponentially bounded*, *there is a constant* $C_{0}=\frac{1}{\alpha}$, $C_{1}=\frac{1}{\alpha}\|A\|^{\frac{1-\alpha}{\alpha}}$, $C_{2}=\frac {1}{\alpha}\|A\|^{\frac{2-\alpha}{\alpha}}$, $e_{\alpha}(t)=e^{\|A\| ^{\frac{1}{\alpha}}t}$, $M=e_{\alpha}(b)$,

3 $$ \bigl\Vert S_{\alpha,1}(A,t)\bigr\Vert \leq C_{0} e_{\alpha}(t),\qquad \bigl\Vert S_{\alpha,\alpha}(A,t) \bigr\Vert \leq C_{1}e_{\alpha}(t). $$

## Open and closed-loop case

In this section, we consider the following fractional equation: $k=0,1,2,3,\ldots$ ,

4 $$ \left \{ \textstyle\begin{array}{l} {}^{\mathrm{c}}\mathrm{D}^{\alpha}_{t} x_{k}(t)=Ax_{k}(t)+Bu_{k}(t),\quad t\in J=[0,b], \\ y_{k}(t)=Cx_{k}(t). \end{array}\displaystyle \right . $$

For equation (4), we apply the following open and closed-loop P-type ILC algorithm, $t\in[0,b]$:

5 $$ u_{k+1}(t)=u_{k}(t)+ L_{1}e_{k}(t)+L_{2}e_{k+1}(t), $$

where $L_{1}$, $L_{2}$ are the parameters which will be determined, $e_{k}=y_{d}(t)-y_{k}(t)$, $y_{d}(t)$ are the given functions. The initial state of each iterative learning is

6 $$ x_{k+1}(0)=x_{k}(0)+ B L_{1}e_{k}(t). $$

We make the following assumptions:

1. $1-\lambda^{-1}C_{1}M\|C\|\|L_{2}B\|>0$,
2. $\frac{\|I-C S_{\alpha,1}(A,t)B L_{1}\|+\lambda^{-1}C_{1}M\| C\|\|L_{1}B\|}{1-\lambda^{-1}C_{1}M\|C\|\|L_{2}B\|}<1$.

### Theorem 3.1

*Assume that the open and closed*-*loop P*-*type ILC algorithm* (5) *is used*, (H1) *and* (H2) *hold*, *let* $y_{k}(\cdot)$ *be the output of equation* (4), *if the initial state of each iterative learning satisfy* (6), $\lim_{k\to\infty}\| e_{k}\|_{\lambda}=0$, $t\in J$.

### Proof

According to (2), (5), and (6), we know

$$\begin{aligned} \begin{aligned} x_{k+1}(t)={}& S_{\alpha,1}(A,t)x_{k+1}(0)+ \int_{0}^{t}S_{\alpha,\alpha }(A,t-s)Bu_{k+1}(s) \,ds \\ ={}&S_{\alpha,1}(A,t) \bigl(x_{k}(0)+ B L_{1}e_{k}(t) \bigr) \\ &{}+ \int_{0}^{t}S_{\alpha,\alpha}(A,t-s)B \bigl(u_{k}(s)+ L_{1}e_{k}(s)+L_{2}e_{k+1}(s) \bigr)\,ds \\ ={}&x_{k}(t)+S_{\alpha,1}(A,t) B L_{1}e_{k}(t) + \int_{0}^{t}S_{\alpha ,\alpha}(A,t-s)BL_{1}e_{k}(s) \,ds \\ &{}+ \int_{0}^{t}S_{\alpha,\alpha}(A,t-s)BL_{2}e_{k+1}(s) \,ds, \end{aligned} \end{aligned}$$

so the $(k+1)$th iterative error is

7 $$\begin{aligned} e_{k+1}(t) =&y_{d}(t)-Cx_{k+1}(t) \\ =&y_{d}(t)-C \biggl(x_{k}(t)+S_{\alpha,1}(A,t) B L_{1}e_{k}(t) + \int _{0}^{t}S_{\alpha,\alpha}(A,t-s)BL_{1}e_{k}(s) \,ds \\ &{}+ \int_{0}^{t}S_{\alpha,\alpha}(A,t-s)BL_{2}e_{k+1}(s) \,ds \biggr) \\ =&e_{k}(t)-C \biggl(S_{\alpha,1}(A,t) B L_{1}e_{k}(t) + \int _{0}^{t}S_{\alpha,\alpha}(A,t-s)BL_{1}e_{k}(s) \,ds \\ &{}+ \int_{0}^{t}S_{\alpha,\alpha}(A,t-s)BL_{2}e_{k+1}(s) \,ds \biggr) \\ =& \bigl(I-C S_{\alpha,1}(A,t)B L_{1} \bigr)e_{k}(t)-C \int_{0}^{t}S_{\alpha ,\alpha}(A,t-s)BL_{1}e_{k}(s) \,ds \\ &{}-C \int_{0}^{t}S_{\alpha,\alpha}(A,t-s)BL_{2}e_{k+1}(s) \,ds; \end{aligned}$$

take the norm of (7),

8 $$\begin{aligned} \bigl\Vert e_{k+1}(t)\bigr\Vert \leq&\bigl\Vert I-C S_{\alpha,1}(A,t)B L_{1}\bigr\Vert \bigl\Vert e_{k}(t)\bigr\Vert \\ &{}+\Vert C\Vert \int_{0}^{t}\bigl\Vert C_{1} e_{\alpha}(s)\bigr\Vert \Vert BL_{1}\Vert \bigl\Vert e_{k}(s)\bigr\Vert \,ds \\ &{}+\Vert C\Vert \int_{0}^{t}\bigl\Vert C_{1} e_{\alpha}(s)\bigr\Vert \Vert BL_{2}\Vert \bigl\Vert e_{k+1}(s)\bigr\Vert \,ds, \end{aligned}$$

take the *λ*-norm of (8),

9 $$\begin{aligned} \Vert e_{k+1}\Vert _{\lambda} \leq&\bigl\Vert I-C S_{\alpha ,1}(A,t)BL_{1}\bigr\Vert \Vert e_{k} \Vert _{\lambda} \\ &{}+\sup_{ t\in[0,T]}e^{-\lambda t}\Vert CC_{1}BL_{1} \Vert \int _{0}^{t}\bigl\Vert e_{\alpha}(s)\bigr\Vert \bigl\Vert e_{k}(s)\bigr\Vert \, ds \\ &{}+\sup_{ t\in[0,T]}e^{-\lambda t}\Vert C\Vert \int_{0}^{t}\bigl\Vert C_{1} e_{\alpha}(s)\bigr\Vert \Vert BL_{2}\Vert \bigl\Vert e_{k+1}(s)\bigr\Vert \,ds \\ \leq&\bigl\Vert I-C S_{\alpha,1}(A,t)BL_{1}\bigr\Vert \Vert e_{k}\Vert _{\lambda} \\ &{}+\sup_{ t\in[0,T]}e^{-\lambda t}\Vert CC_{1}BL_{1} \Vert \int _{0}^{t}\bigl\Vert e_{\alpha}(s)\bigr\Vert e^{\lambda s}\,ds\Vert e_{k}\Vert _{\lambda} \\ &{}+\sup_{ t\in[0,T]}e^{-\lambda t}\Vert C\Vert \int_{0}^{t}\bigl\Vert C_{1} e_{\alpha}(s)\bigr\Vert \Vert BL_{2}\Vert e^{\lambda s} \,ds\Vert e_{k+1}\Vert _{\lambda}, \end{aligned}$$

if $1-\lambda^{-1}C_{1}M\|C\|\|L_{2}B\|>0$,

10 $$ \| e_{k+1}\|_{\lambda}\leq\frac{\|I-C S_{\alpha,1}(A,t)B L_{1}\| +\lambda^{-1}C_{1}M\|C\|\|L_{1}B\|}{1-\lambda^{-1}C_{1}M\|C\|\|L_{2}B\|}\| e_{k}\|_{\lambda}, $$

let $\frac{\|I-C S_{\alpha,1}(A,t)B L_{1}\|+\lambda^{-1}C_{1}L_{1}M\|C\| \|B\|}{1-\lambda^{-1}C_{1}L_{2}M\|C\|\|B\|}<1$, (10) is a contraction mapping, and it follows from the contraction mapping that $\lim_{k\to\infty}\| e_{k}\|_{\lambda}=0$, $t\in J$. This completes the proof. □

Theorem 3.1 implied that the tracking error $e_{k}(t)$ depends on *C* and $x_{k}(t)$, it is also observed for (9) that the boundedness of the parameters *C*, *B*, $L_{0}$, $L_{1}$ implies the boundedness of the $\|e_{k}\|_{\lambda}$, so Theorem 3.1 indirectly indicated that the output error also depend on $\frac{\|I-C S_{\alpha,1}(A,t)B L_{1}\|+\lambda^{-1}C_{1}M\|C\|\|L_{1}B\|}{1-\lambda ^{-1}C_{1}M\|C\|\|L_{2}B\|}$. From the result, we can do a more in-depth discussion.

### Corollary 3.2

*Suppose that all conditions are the same with Theorem* 3.1, $\lim_{k\to\infty}\| e_{k}\|_{\lambda}=0$, *then*

$$\frac{\ln{\frac{C_{1}M\|L_{1}\|+C_{1}M\|L_{2}\|}{\lambda C_{0}\|L_{1}\| }}}{\| A\|^{\frac{1}{\alpha}}}< t< b. $$

### Proof

From Theorem 3.1, the important condition is $\frac{\|I-C S_{\alpha,1}(A,t)B L_{1}\|+\lambda^{-1}C_{1}M\|C\|\|L_{1}B\|}{1-\lambda ^{-1}C_{1}M\|C\|\|L_{2}B\|}<1$, which implies that

$$1-C_{0} e_{\alpha}(t)\|C\| \|L_{1}B \|+ \lambda^{-1}C_{1}M\|C\|\|L_{1}B\| \leq1- \lambda^{-1}C_{1}M\|C\|\|L_{2}B\|, $$

we can get

$$\frac{\ln{\frac{C_{1}M\|L_{1}\|+C_{1}M\|L_{2}\|}{\lambda C_{0}\|L_{1}\| }}}{\| A\|^{\frac{1}{\alpha}}}< t< b. $$

 □

## P-type ILC for some fractional system with random disturbance

In this section, we consider the following fractional equation: $k=0,1,2,3,\ldots$ ,

11 $$ \left \{ \textstyle\begin{array}{l} {}^{\mathrm{c}}\mathrm{D}^{\alpha}_{t} x_{k}(t)=Ax_{k}(t)+Bu_{k}(t)+\omega _{k}(t),\quad t\in J=[0,b], \\ y_{k}(t)=Cx_{k}(t)+\nu_{k}(t), \end{array}\displaystyle \right . $$

where $\omega_{k}(t)$, $\nu_{k}(t)$ are the random disturbance.

Firstly, we will make some assumptions to be satisfied on the data of our problem:

(H3): $\|\omega_{k} \|_{\lambda}\leq\varepsilon_{1}$, $\|\nu_{k} \| _{\lambda}\leq\varepsilon_{2}$ for some positive constants $\varepsilon _{1}$, $\varepsilon_{2}$,
(H4): $\rho_{1}=\|I+CS_{\alpha,1}(A,t)L_{2} B \|-\lambda^{-1}C_{1}\| C\|\|L_{2}B\|M>0$, $\rho_{2}=\|I-CS_{\alpha,1}(A,t)L_{1} B \|+\lambda^{-1}C_{1}\|C\|\|L_{1}B\|M$.

For equation (11), we choose the following open and closed-loop P-type ILC algorithm, $t\in[0,b]$:

12 $$ u_{k+1}(t)=u_{k}(t)+ L_{1}e_{k}(t)+L_{2}e_{k+1}(t), $$

where $L_{1}$, $L_{2}$ are the parameters which will be determined, $e_{k}=y_{d}(t)-y_{k}(t)$, $y_{d}(t)$ are the given functions.

Assume that the initial state of each iterative learning is (13), where $L_{1}$, $L_{2}$ are the parameters which will be determined. We have

13 $$ x_{k+1}(0)=x_{k}(0)+ B L_{1}e_{k}(t)+B L_{2}e_{k+1}(t). $$

### Theorem 4.1

*Assume that the hypotheses* (H3), (H4) *are satisfied*, *let* $y_{k}(\cdot)$ *be the output of equation* (2), *if* $\varepsilon_{1}\rightarrow0$ *and* $\varepsilon_{2}\rightarrow0$, $\rho _{1}>\rho_{2}$, *the open and closed*-*loop P*-*type ILC* (12) *guarantees that* $\lim_{k\to\infty}\| e_{k}\|_{\lambda}=0$, $t\in J$.

### Proof

According to (2) and assumptions (H2), (H3), we know

$$\begin{aligned} x_{k+1}(t) =& S_{\alpha,1}(A,t)x_{k+1}(0)+ \int_{0}^{t}S_{\alpha,\alpha}(A,t-s) \bigl(Bu_{k+1}(s)+\omega_{k+1}(s) \bigr) \,ds \\ =&S_{\alpha,1}(A,t) \bigl(x_{k}(0)+ B L_{1}e_{k}(t)+B L_{2}e_{k+1}(t) \bigr) \\ &{}+ \int_{0}^{t}S_{\alpha,\alpha}(A,t-s)B \bigl(u_{k}(s)+ L_{1}e_{k}(s)+L_{2}e_{k+1}(s) \bigr)\,ds \\ &{}+ \int_{0}^{t}S_{\alpha,\alpha}(A,t-s) \omega_{k+1}(s)\,ds \\ =&x_{k}(t)+S_{\alpha,1}(A,t) B L_{1}e_{k}(t)+ S_{\alpha,1}(A,t) B L_{2}e_{k+1}(t) \\ &{}+ \int_{0}^{t}S_{\alpha,\alpha}(A,t-s)BL_{1}e_{k}(s) \,ds + \int _{0}^{t}S_{\alpha,\alpha}(A,t-s)BL_{2}e_{k+1}(s) \,ds \\ &{}+ \int_{0}^{t}S_{\alpha,\alpha}(A,t-s) \omega_{k+1}(s)\,ds, \end{aligned}$$

the $(k+1)$th iterative error is

14 $$\begin{aligned} e_{k+1}(t) =&y_{d}(t)-Cx_{k+1}(t)- \nu_{k+1}(t) \\ =&y_{d}(t)-C \biggl(x_{k}(t)+S_{\alpha,1}(A,t) B L_{1}e_{k}(t)+S_{\alpha ,1}(A,t) B L_{2}e_{k+1}(t) \\ &{}+ \int_{0}^{t}S_{\alpha,\alpha}(A,t-s)BL_{1}e_{k}(s) \,ds+ \int _{0}^{t}S_{\alpha,\alpha}(A,t-s)BL_{2}e_{k+1}(s) \,ds \\ &{}+ \int_{0}^{t}S_{\alpha,\alpha}(A,t-s) \omega_{k+1}(s)\,ds \biggr)-\nu _{k+1}(t) \\ =&e_{k}(t)-C \biggl(S_{\alpha,1}(A,t) B L_{1}e_{k}(t)+S_{\alpha,1}(A,t) B L_{2}e_{k+1}(t) \\ &{}+ \int_{0}^{t}S_{\alpha,\alpha}(A,t-s)BL_{1}e_{k}(s) \,ds+ \int _{0}^{t}S_{\alpha,\alpha}(A,t-s)BL_{2}e_{k+1}(s) \,ds \\ &{}+ \int_{0}^{t}S_{\alpha,\alpha}(A,t-s) \omega_{k+1}(s)\,ds \biggr)-\nu _{k+1}(t) \\ =& \bigl(I-C S_{\alpha,1}(A,t)B L_{1} \bigr)e_{k}(t)-CS_{\alpha,1}(A,t) B L_{2}e_{k+1}(t) \\ &{}-C \int_{0}^{t}S_{\alpha,\alpha}(A,t-s)BL_{1}e_{k}(s) \,ds-C \int _{0}^{t}S_{\alpha,\alpha}(A,t-s)BL_{2}e_{k+1}(s) \,ds \\ &{}-C \int_{0}^{t}S_{\alpha,\alpha}(A,t-s) \omega_{k+1}(s)\,ds-\nu_{k+1}(t). \end{aligned}$$

Taking the norm of (14), it is easy to obtain

15 $$\begin{aligned}& \bigl\Vert I+CS_{\alpha,1}(A,t)L_{2} B \bigr\Vert \bigl\Vert e_{k+1}(t)\bigr\Vert \\& \quad\leq\bigl\Vert I-C S_{\alpha,1}(A,t)L_{1}B\bigr\Vert \bigl\Vert e_{k}(t)\bigr\Vert +\Vert C\Vert \int_{0}^{t}\bigl\Vert C_{1} e_{\alpha }(s)\bigr\Vert \Vert L_{1}B\Vert \bigl\Vert e_{k}(s)\bigr\Vert \,ds \\& \qquad{}+\Vert C\Vert \int_{0}^{t}\bigl\Vert C_{1} e_{\alpha}(s)\bigr\Vert \Vert L_{2}B\Vert \bigl\Vert e_{k+1}(s)\bigr\Vert \,ds+\Vert C\Vert \int _{0}^{t}\bigl\Vert C_{1} e_{\alpha}(s)\bigr\Vert \bigl\Vert \omega_{k+1}(s)\bigr\Vert \,ds \\& \qquad{}+\bigl\Vert \nu_{k+1}(t)\bigr\Vert , \end{aligned}$$

once more using the *λ*-norm, we have

$$\begin{aligned}& \bigl\Vert I+CS_{\alpha,1}(A,t)L_{2} B \bigr\Vert \Vert e_{k+1}\Vert _{\lambda}\\& \quad\leq\bigl\Vert I-C S_{\alpha,1}(A,t)L_{1}B\bigr\Vert \Vert e_{k}\Vert _{\lambda}+\sup_{ t\in[0,T]}e^{-\lambda t}C_{1} \Vert C\Vert \Vert BL_{1}\Vert \int_{0}^{t}\bigl\Vert e_{\alpha}(s)\bigr\Vert e^{\lambda s}\, ds\Vert e_{k}\Vert _{\lambda}\\& \qquad{}+\sup_{ t\in[0,T]}e^{-\lambda t}C_{1}\Vert C \Vert \Vert L_{2}B\Vert \int_{0}^{t}\bigl\Vert e_{\alpha}(s)\bigr\Vert e^{\lambda s}\, ds\Vert e_{k+1}\Vert _{\lambda}\\& \qquad{}+\sup_{ t\in[0,T]}e^{-\lambda t}C_{1}\Vert C \Vert \int _{0}^{t}\bigl\Vert e_{\alpha}(s)\bigr\Vert e^{\lambda s}\,ds\Vert \omega _{k+1}\Vert _{\lambda}+\sup_{ t\in[0,T]}e^{-\lambda t}\bigl\Vert \nu_{k+1}(t) \bigr\Vert , \end{aligned}$$

invoking (H3) and (H4), if $\varepsilon=\lambda^{-1}C_{1}\varepsilon_{1}M\| C\|+\varepsilon_{2}$,

16 $$ \rho_{1} \| e_{k+1}\|_{\lambda}\leq \rho_{2}\|e_{k}\|_{\lambda}+\varepsilon, $$

which implies that

$$\| e_{k}\|_{\lambda}\leq\frac{\varepsilon}{\rho_{1}-\rho_{2}}, $$

if $\varepsilon_{1}\rightarrow0$ and $\varepsilon_{2}\rightarrow0$, $\varepsilon\rightarrow0$, thus $\lim_{k\to\infty}\| e_{k}\|_{\lambda}=0$, $t\in J$, and this completes the proof. □

From Theorem 4.1, on the one hand, the random disturbance makes some impact on the system (11), $\varepsilon _{1}\rightarrow0$ and $\varepsilon_{2}\rightarrow0$ imply the impact is very small; on the other hand, $\rho_{1}>\rho_{2}$, for this condition, we illustrate the following corollary.

### Corollary 4.2

*Suppose that all conditions are the same as Theorem* 4.1, $\lim_{k\to\infty}\| e_{k}(t)\|_{\lambda}=0$, *then* *t* *satisfies*

$$\frac{\ln|\frac{C_{1}M}{\lambda C_{0}}|}{\|A\|^{\frac{1}{\alpha}}}< t < \frac{\ln|\frac{1-\lambda^{-1}C_{1}\|C\|\|L_{2}B\|M}{\|C\|\|L_{2}B\|}|}{\|A\| ^{\frac{1}{\alpha}}}. $$

### Proof

According to (H4), $\|I+CS_{\alpha,1}(A,t)L_{2} B \|-\lambda ^{-1}C_{1}\|C\|\|L_{2}B\|M>0$, then

$$t< \frac{\ln|\frac{1-\lambda^{-1}C_{1}\|C\|\|L_{2}B\|M}{\|C\|\|L_{2}B\|}|}{\| A\|^{\frac{1}{\alpha}}}. $$

From Theorem 4.1, we know that $\varepsilon_{1}\rightarrow0$ and $\varepsilon_{2}\rightarrow0$, and the condition is

$$\frac{\rho_{2}}{\rho_{1}}=\frac{\|I-CS_{\alpha,1}(A,t)L_{1} B \|+\lambda ^{-1}C_{1}\|C\|\|L_{1}B\|M}{\|I+CS_{\alpha,1}(A,t)L_{2} B \|-\lambda ^{-1}C_{1}\|C\|\|L_{2}B\|M}< 1, $$

which yields $\frac{\ln|\frac{C_{1}M}{\lambda C_{0}}|}{\|A\|^{\frac {1}{\alpha}}}< t $. At last, we obtain the estimate

$$\frac{\ln|\frac{C_{1}M}{\lambda C_{0}}|}{\|A\|^{\frac{1}{\alpha}}}< t < \frac{\ln|\frac{1-\lambda^{-1}C_{1}\|C\|\|L_{2}B\|M}{\|C\|\|L_{2}B\|}|}{\|A\| ^{\frac{1}{\alpha}}}. $$

 □

## Simulations

In this section, we will give two simulation examples to demonstrate the validity of the algorithms.

### P-type ILC with initial state error

17 $$ \left \{ \textstyle\begin{array}{l} {}^{\mathrm{c}}\mathrm{D}^{0.5}_{t} x_{k}(t)=x_{k}(t)+0.2u_{k}(t), \quad t\in J=[0,1.8], \\ x(0)=0.5, \\ y_{k}(t)=x_{k}(t), \end{array}\displaystyle \right . $$

with the iterative learning control and initial state error

18 $$ \left \{ \textstyle\begin{array}{l} u _{k+1}(t)=u _{k}(t)+ 0.5e_{k}(t)+ 0.5e_{k+1}(t), \\ x _{k+1}(0)=x _{k}(0)+0.1 e_{k}(t). \end{array}\displaystyle \right . $$

We set the initial control $u_{0}(\cdot)=0$, $y_{d}(t)=3t^{2}(1-5t)$, $t\in(0,1.8)$, and set $\alpha=0.5$, $A=1$, $B=0.2$, $C=0.5$, $\lambda =2$, $L_{1}=L_{2}=0.5$, and $C_{0}=2$, $C_{1}=2$, $\lambda-\|A\|^{\frac {1}{\alpha}}=1>0$, $M\approx6>0$, $1-\lambda^{-1}C_{1}M\|C\|\|L_{2}B\| \approx0.7>0$, $\frac{\|I-C S_{\alpha,1}(A,t)B L_{1}\|+\lambda ^{-1}C_{1}M\|C\|\|L_{1}B\|}{1-\lambda^{-1}C_{1}M\|C\|\|L_{2}B\|}\approx \frac{0.5}{0.7}<1$, all conditions of Theorem 3.1 are satisfied.

**Figure 1:**
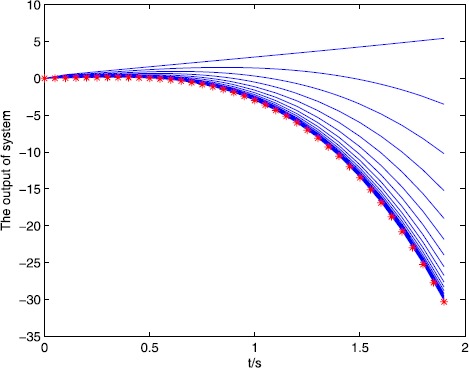
$\pmb{{\ast}{\ast}{\ast}}$ **denotes the desired trajectory, — denotes the output of the system.**

**Figure 2:**
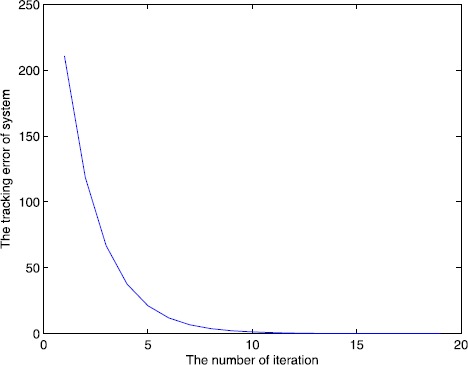
**Number of iterations and tracking error.**

The simulation result can be seen from Figure 1 and Figure 2, for the open and closed-loop P-type ILC system (17), with the increase of the number of iterations, it can track the desired trajectory gradually by using the algorithm. We do not use the single iteration rate to get the result, because in the late of the iteration, the output of the system may jump around the desired trajectory, so we adopt a correction method, that is, when $e(k)>0$, $u(k)=u(k)-0.5\times e(k)$ or $e(k)<0$, $u(k)=u(k)+0.5\times e(k) $, *k* is the number of iteration, the result approaches the desired trajectory stably and quickly, from Figure 2, the tracking error tends to zero at the 15th iteration, so the iterative learning control is feasible and the efficiency is high.

### P-type ILC with random disturbance

Consider the following P-type ILC system:

19 $$ \left \{ \textstyle\begin{array}{l} {}^{\mathrm{c}}\mathrm{D}^{0.5}_{t} x_{k}(t)=x_{k}(t)+0.2u_{k}(t)+10^{-15}t, \quad t\in J=[0,1.8], \\ x(0)=0.5, \\ y_{k}(t)=0.5x_{k}(t)+10^{-10}t^{2}, \end{array}\displaystyle \right . $$

with the iterative learning control and initial state error

20 $$ \left \{ \textstyle\begin{array}{l} u _{k+1}(t)=u _{k}(t)+ e_{k}(t)+ 0.5e_{k+1}(t), \\ x _{k+1}(0)=x _{k}(0)+0.2 e_{k}(t)+0.1e_{k+1}(t). \end{array}\displaystyle \right . $$

**Figure 3:**
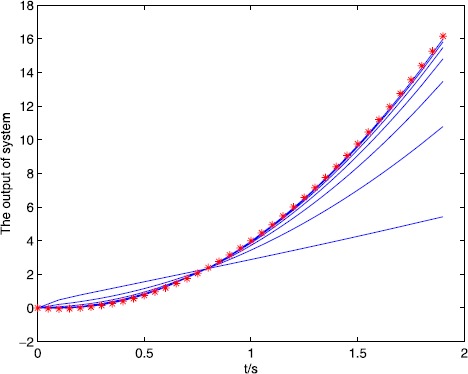
$\pmb{{\ast}{\ast}{\ast}}$ **denotes the desired trajectory, — denotes the output of the system.**

**Figure 4:**
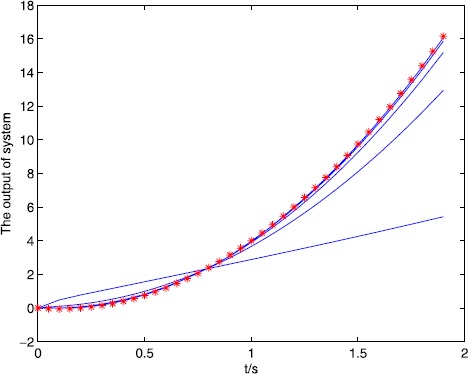
$\pmb{{\ast}{\ast}{\ast}}$ **denotes the desired trajectory, — denotes the output of the system.**

**Figure 5:**
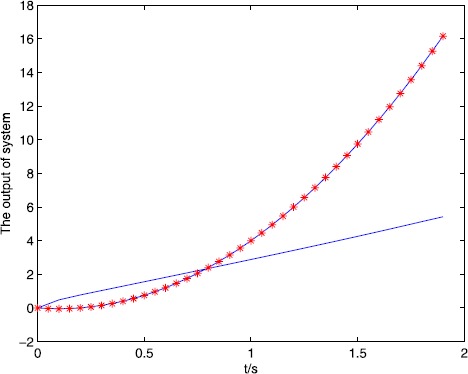
$\pmb{{\ast}{\ast}{\ast}}$ **denotes the desired trajectory, — denotes the output of the system.**

**Figure 6:**
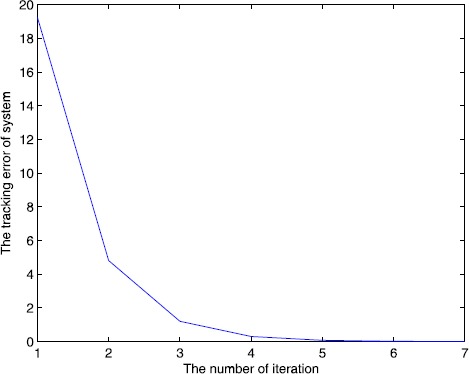
**Number of iterations and tracking error.**

**Figure 7:**
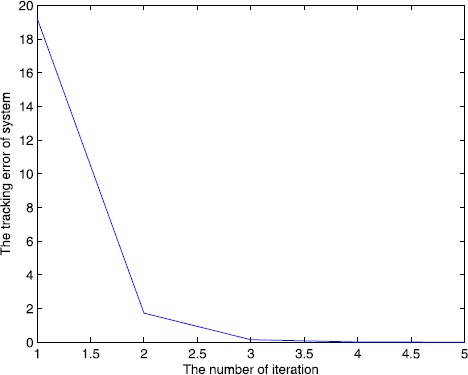
**Number of iterations and tracking error.**

**Figure 8:**
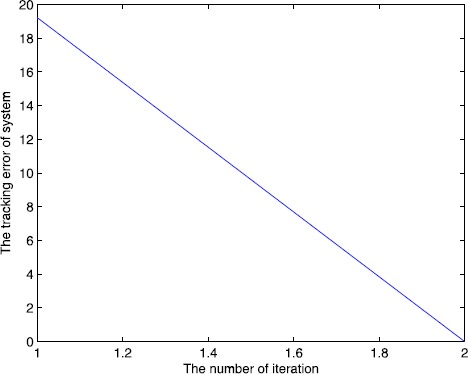
**Number of iterations and tracking error.**

We set the initial control $u_{0}(\cdot)=0$, $y_{d}(t)=5t^{2}-t$, $t\in (0,1.8)$, and set $\alpha=0.5$, $A=1$, $B=0.2$, $C=1$, $\lambda=2$, $L_{1}=1$, $L_{2}=0.5$, and $C_{0}=2$, $C_{1}=2$, $\rho_{1}\approx1.65$, $\rho _{2}\approx0.65$, $\varepsilon_{1}=10^{-15}\rightarrow0$, $\varepsilon _{2}=10^{-10}\rightarrow0$, all conditions of Theorem 4.1 are satisfied. We also use a correction method, that is, when $e(k)>0$, $u(k)=u(k)-m\times e(k)$ or $e(k)<0$, $u(k)=u(k)+m\times e(k) $, *k* is the number of iterations, m is the parameter, we set $m=0.5, 0.7, 1$, and the output of the system is shown in Figure 3, Figure 4, Figure 5. The symbol ∗∗∗ denotes the desired trajectory, — denotes the output of the system, the tracking error is shown in Figure 6, Figure 7, Figure 8, which imply the number of iteration and the tracking error.

From Figures 3-8 and Table 1, we find the tracking error tends to zero within 7 iterations, so the output of the system can track the desired trajectory almost perfectly. By comparing three cases, when $m=1$, the iteration number is only 2, and the tracking error is 0.0001, thus the tracking performance is best and improved over the iteration domain.

**Table 1:** **The iteration number and the tracking error and the running time table**

***m***	**The number of iterations**	**The tracking error**	**Run time (second)**
0.5	7	0.002	58.207
0.7	5	0.0013	50.123
1	2	0.0001	24.844
